# Evolutionary conservation of plant gibberellin signalling pathway components

**DOI:** 10.1186/1471-2229-7-65

**Published:** 2007-11-29

**Authors:** Filip Vandenbussche, Ana C Fierro, Gertrud Wiedemann, Ralf Reski, Dominique Van Der Straeten

**Affiliations:** 1 Unit Plant Hormone Signaling & Bio-imaging, Department of Molecular Genetics, Ghent University, Ledeganckstraat 35, B-9000 Ghent, Belgium; 2 Department Microbial and Molecular Systems, K.U. Leuven, Kasteelpark Arenberg 20, 3000 Leuven, Belgium; 3 Plant Biotechnology, Faculty of Biology, University of Freiburg, Schaenzlestr. 1, 79104 Freiburg, Germany

## Abstract

**Background:**

Gibberellins (GA) are plant hormones that can regulate germination, elongation growth, and sex determination. They ubiquitously occur in seed plants. The discovery of gibberellin receptors, together with advances in understanding the function of key components of GA signalling in Arabidopsis and rice, reveal a fairly short GA signal transduction route. The pathway essentially consists of GID1 gibberellin receptors that interact with F-box proteins, which in turn regulate degradation of downstream DELLA proteins, suppressors of GA-controlled responses.

**Results:**

*Arabidopsis *sequences of the gibberellin signalling compounds were used to screen databases from a variety of plants, including protists, for homologues, providing indications for the degree of conservation of the pathway. The pathway as such appears completely absent in protists, the moss *Physcomitrella patens *shares only a limited homology with the Arabidopsis proteins, thus lacking essential characteristics of the classical GA signalling pathway, while the lycophyte *Selaginella moellendorffii *contains a possible ortholog for each component. The occurrence of classical GA responses can as yet not be linked with the presence of homologues of the signalling pathway. Alignments and display in neighbour joining trees of the GA signalling components confirm the close relationship of gymnosperms, monocotyledonous and dicotyledonous plants, as suggested from previous studies.

**Conclusion:**

Homologues of the GA-signalling pathway were mainly found in vascular plants. The GA signalling system may have its evolutionary molecular onset in *Physcomitrella patens*, where GAs at higher concentrations affect gravitropism and elongation growth.

## Background

Gibberellins (GAs) are a large family of hormones that are important for a vast array of responses throughout the life cycle of plants. They mainly stimulate germination, cause cell expansion, and regulate flowering time. Due to their high economical relevance, the effects of GAs on cell elongation are subject to intense scientific studies. The green revolution was based on selection for dwarfism in rice and wheat cultivars. Recently it was shown that these dwarfing genes interfere with either the production or the action of GAs [1]. Chemical interference with GA biosynthesis is often used to limit the growth of plants, including trees [2]. GAs were first isolated from *Gibberella *(*Fusarium*) *fujikuroi *[3]. This fungus causes extreme extension growth in rice, named bakanae or "foolish" rice, which hence is far more susceptible to lodging.

Apart from *Gibberella*, other fungi (*Phaeospheria*, *Aphaceloma *sp.) and various bacteria [4] are able to synthesize GAs. GAs were consequently found in many plant species and are widespread over photosynthesizing organisms. GA-like substances were detected in unicellular and multicellular algae [5-7], in lichens and mosses [8], and in ferns [4] But most of all, they are widely accepted as general growth controlling hormones in seed plants [4].

Presence of GAs in an organism does not necessarily mean that it is responsive to these compounds. For instance, *Gibberella *itself does not react to exogenous GA [3]. Depending on the species of unicellular algae, GA can slightly increase the biomass [9]. The effects of GAs on elongation growth of unicellular algae are either very small or absent in most species [10]. However, growth increases were reported for the multicellular alga *Porphyra *in the diploid, filamentous sporophyte conchocelis phase and, in combination with auxin, in stolons of *Ulva lactuca *[10,11].

While ABA, auxin, and cytokinin induce specific developmental alterations in mosses like *Physcomitrella patens*, no such effects have been reported for GA-application [12,13]. However, some older reports do exist, that GA-application on specific moss species may slightly enhance growth rates [14,15]. In addition GA-application may interfere with gravitropism in the mosses *Ceratodon purpureus *[16] and *Pottia intermedia *[17]. In fact, to date gibberellins have not been identified in mosses, and it was proposed that the hormonal signalling pathway developed later in land plant evolution [13]. However, as such pathways do not appear completely *de novo*, precursors from which GA may have been evolved should be present in mosses. Ent-kaurene is the key intermediate in the biosynthesis of gibberellins. Recently, it was shown, that *P. patens *produces as a secondary metabolite such a tetracyclic diterpene as a volatile compound in huge amounts [18], and possesses a bifunctional ent-kaurene synthase [19].

To our knowledge, no reports are available for GA regulated growth in ferns. In contrast, their stimulatory effect on elongation and germination has been extensively documented for seed plants, such as conifers [20,21] and angiosperms, both mono- and dicotyledonous plants.

Sex determination is another known GA effect. In species as *Chara*, GA promote antheridia (male sex organ) formation [22]. Likewise, they serve as promoters for antheridia formation in some ferns [23,24]. Interestingly, in many eudicotyledonous plants as well, GAs promote male flower development [25]. By contrast, in the monocot maize, they promote female flower formation [26].

**Figure 1:**
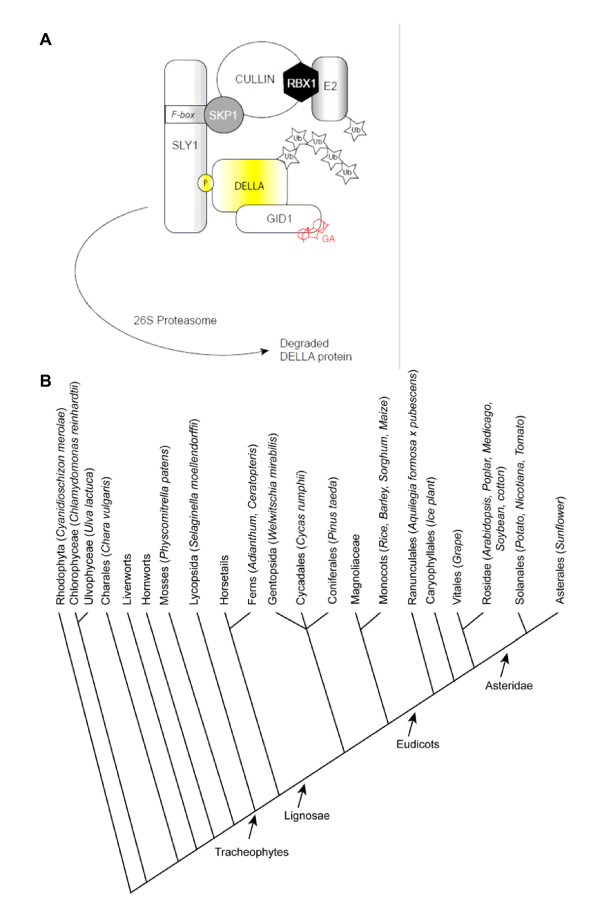
A) Current model for GA signalling. GA is bound to the GID1 GA receptor and which stimulates the interaction with the DELLA transcription factor. The F-box protein SLY1/GID2 connects the DELLA transcription factors to an SCF-E3 ubiquitin ligase. Ubiquitin is transferred from an E2 onto the DELLA target protein. The ensemble of these events causes subsequent rapid degradation by the 26S proteasome. B) Simplified overview of plant evolution based on the hyperbolic tree presented at [62]. Clades that are discussed in the text are retained in the graph. In parenthesis, genera and species mentioned in the text are indicated.

It is speculated that the GA signalling pathway, which ultimately leads to germination and elongation growth in seed plants, consists of a limited number of factors (figure 1A). Recently, GA receptors of rice (GID1) and *Arabidopsis *have been discovered [27,28]. The receptors are able to interact, in a GA dependent way, with the DELLA proteins [27,28]; the interactions solely depends on the presence of the DELLA-domain in the DELLA protein [29]. DELLA proteins (such as RGA in Arabidopsis) are inhibitors of GA responses, which are broken down in the presence of GAs, hence promoting germination and elongation growth [30]. This degradation is mediated by an E3 ligase, that is specified by an F-box protein (such as SLY1 in Arabidopsis or GID2 in rice) [31-33]. Thus the GA signalling pathway essentially consists of three interacting players.

Over the last few years, three plant genomes have been entirely sequenced [34-36] and a lot of effort is made to unravel the gene pool of "genomically more complex" plants by expressed sequenced tag (EST) sequencing and assembly. With the variety of open access EST and genome sequence databases, it becomes possible to perform comparative genomics and molecular phylogenetic analysis on a large number of species at a time (plantGDB, [37]). Based on the aforementioned tools, we investigated the conservation of the GA pathway formed by GID1, SLY1 and DELLA proteins from algae and plants throughout the plant kingdom, using reference species for different major plant groups (Figure 1B). Existing orthologues were retrieved from various higher plants, but are missing from *Physcomitrella patens *(*Pp*). The presence of the GA signalling pathway could thus be linked exclusively with vascular plants.

## Results

### The gibberellin receptors

The GA receptors (GID1) belong to the large family of hormone sensitive lipases and have homologues throughout the plant kingdom, since they are related in sequence to various carboxylesterases [38]. Moreover, the predicted 3D structure of these proteins is similar. However, the GA receptors contain various regions, which distinguish them from their carboxylesterase relatives [28,38]. Amino acid Arg265 is typically present, while the His340 residue from the catalytic site of the esterases is missing in GID1 proteins.

**Figure 2:**
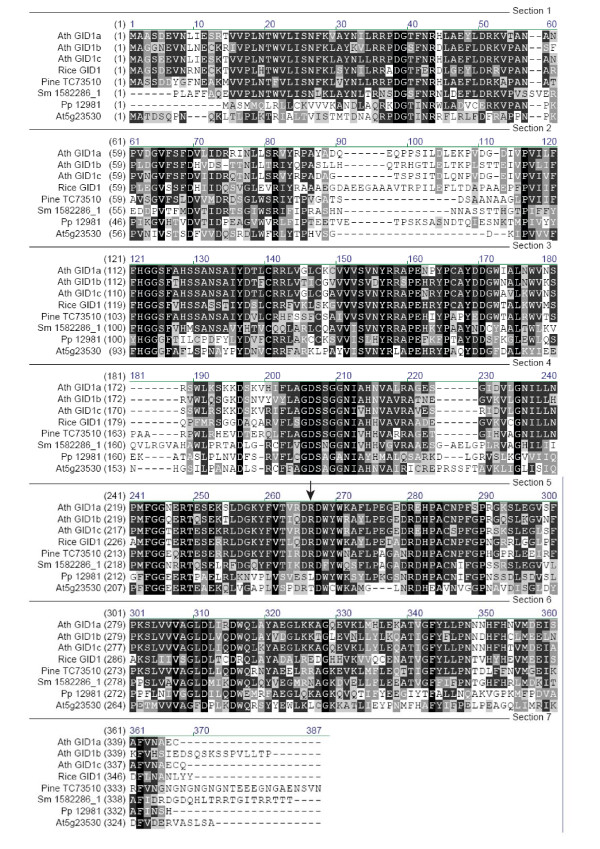
Alignment of GA receptor homologues. The arrow indicates Arg265 which is necessary for GA binding. Ath: *Arabidopsis thaliana*, Sm: *Selaginella moellendorffii*, Pp: *Physcomitrella patens*.

When performing TBLASTN searches, no homologues (E < e-10) were found in *Cyanidioschyzon merolae *(belonging to the phylum Rhodophyta, red algae), nor in *Chlamydomonas reinhardtii *(belonging to the phylum Chlorophyta, amongst the green algae). In the moss *Physcomitrella patens *(*Pp*), the closest homologue to the *Arabidopsis *GA receptors misses the Arg265, it has a Leu instead (Figure 2). This Arg is especially important for gibberellin binding, as proteins carrying the *gid1-2 *mutation, where this residue is changed into a Thr, are unable to bind gibberellins in vitro [27]. Furthermore, the N and C-terminal end of the *Physcomitrella *protein diverge from the GID1 proteins. The protein encoded by AT5G23530 also lacks Arg265, which is unique in the GA receptors, but retains the His340 from the catalytic site of the esterases, which is replaced by an Ile or Leu in the GA receptors and a Trp in the *Pp *homologue. These data support the idea that the closest homologue in *Pp *is part of the large carboxylesterase-like gene family, but lacks features essential for full gibberellin signalling and features conserved in esterases.

The seedless vascular plant *Selaginella moellendorffii *(belonging to the phylum of Lycopodiophyta) has a protein that appears a genuine GA receptor orthologue. It has an overall good aligment (47% similarity) and in contrast to the protein from *Pp*, it contains the Arg265 residue. It also has a conserved Ile in position 340 instead of a His, typical for known GA receptors.

From the analysis of a limited EST set (~8000) of the fern *Adianthum capillus veneris *(phylum Pteridophyta, order Polypodiales), we concluded that it has related carboxylesterases, all with the conserved His340 necessary for esterase activity. Yet the EST collection is too small to firmly exclude the existence of genuine GA receptor orthologues.

**Figure 3:**
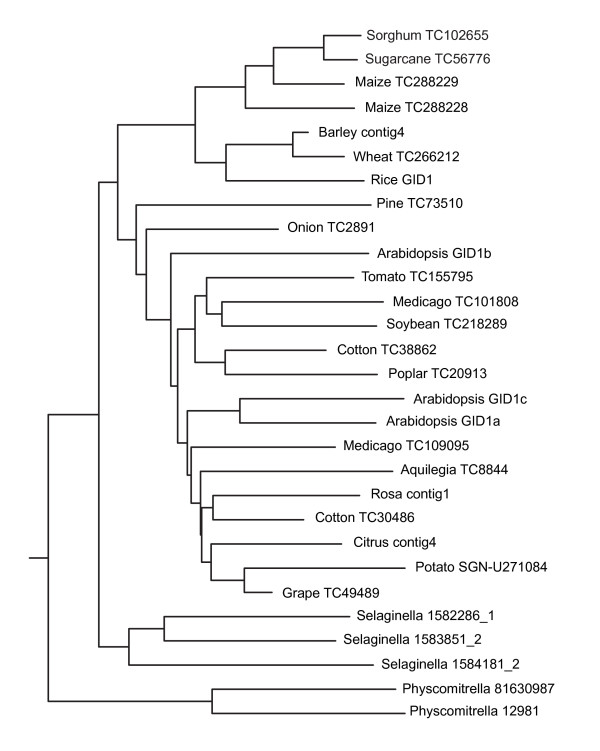
Neighbour-Joining tree of DNA sequences homologous to that encoding the Arabidopsis GID1b GA receptor.

In seed plants, including gymnosperms and angiosperms, GID1 orthologues are widely present. A clear separation between monocots and eudicots is visible in a Neighbour-Joining (N-J) tree (Figure 3). Interestingly, the pine homologue falls within the various sequences from dicots, suggesting a high conservation for this protein among higher plants. On the whole, the ClustalW alignment based N-J tree of the GA receptors shows a relation that is highly reminiscent of the evolutionary schemes generally accepted today [39], with the overall most related plants clustering together.

### The SLEEPY homologues

No (E < 1e^-4^) homologues to the F-box protein SLY1 were found in *Chlamydomonas reinhardtii*, nor *Cyanidioschyzon merolae *using TBlastX.

**Figure 4:**
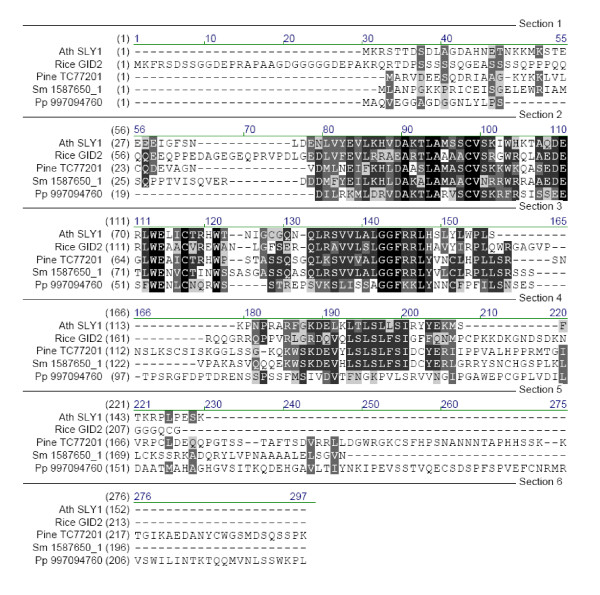
Alignment of the SLY1 homologues. Ath: *Arabidopsis thaliana*, Sm: *Selaginella moellendorffii*, Pp: *Physcomitrella patens*.

A reciprocal blast yielding three other *Arabidopsis *proteins (At3g44326, At2g27310, At2g36090 with BLASTP scores of 71, 56, and 55 respectively) closer related to the *Pp *homologue of SLY1 (997094760 in Figure 4) than SLY1 itself (BLASTP score 54), indicates that SLY1 orthologues are not present in *Pp*. In fact, the highest homology with SLY1, within the *Pp *protein, occurs in the F-box region. This region is necessary for interactions with the Skp1 partner within the SCF-complex, and does not confer substrate specificity. The latter is usually achieved by the C-terminal part [40]. The C-terminal part of the *Pp *homologue is highly divergent from the rice and *Arabidopsis *F-Box proteins GID2 and SLY1 (Figure 4). The GGF (AA positions 120–160 in Figure 4) and LSL motives, conserved in the GID2 and SLY1 [31,41], in the *Pp *homologue, have lower and almost no homology with the GA related F-box proteins. It is therefore questionable and rather unlikely that the *Pp *protein could recognize a DELLA protein as a substrate.

From *Selaginella*, a protein could be retrieved with homology to the SLY1 over its whole length, including a leucine rich box at the C-terminal end (Figure 4). A reciprocal blast indicates that the protein can be considered as an orthologue of those of higher plants (similarity 51%).

The EST database from the fern *Ceratopteris *only yielded an F-box protein with a divergent C-terminal end (data not shown). F-box proteins are thus present in ferns, but SLY1 orthologues remain to be discovered.

**Figure 5:**
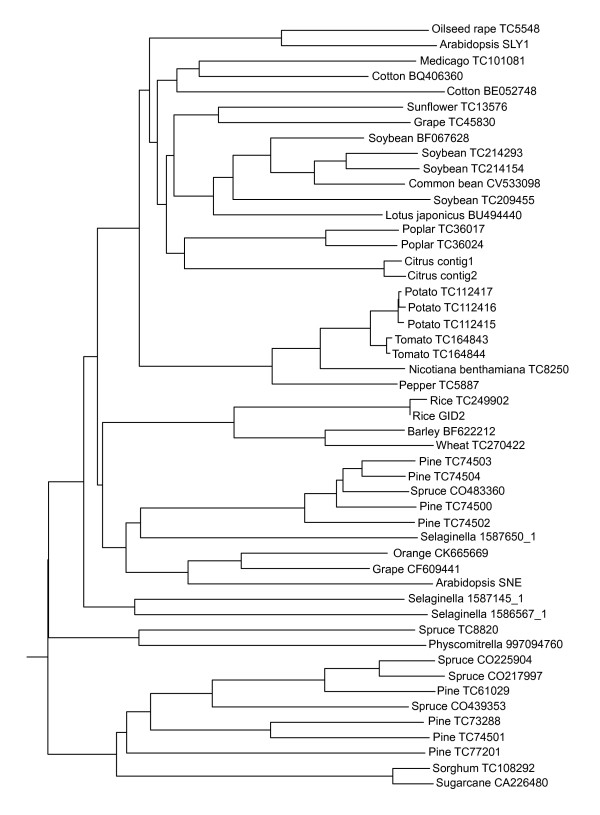
Neighbour-Joining tree of DNA sequences homologous to that encoding the Arabidopsis F-box protein SLY1.

Similarly to the GA receptors, in gymnosperms and angiosperms, SLY1 orthologues are widely present. However, F-box proteins are a very divergent family of polypeptides. The eudicot SLY1 orthologues form a cluster in a N-J tree (Figure 5), while the monocots are grouped with the gymnosperms. There is a distinct group containing *Sorghum*, sugarcane and many conifer sequences. In this group of sequences, the evolutionary distance to SLY1 is larger than that to the *Pp *homologue. A second monocot-gymnosperm cluster groups with the closest SLY1 homologue in *Arabidopsis*, SNEEZY (SNE), and its homologues (Figure 5; [42]). Overexpression of SNE in *Arabidopsis *can take over GA signalling events, but a clear involvement in GA signalling in natural conditions has not been found.

### DELLA related transcription factors

DELLA proteins belong to the GRAS (GAI, RGA, SCARECROW) protein family, specific to plants [43]. The cDNA sequence of RGA1 of *Arabidopsis *was used in a TBLASTX screen for homologues in other species.

No DELLA homologues (yield is 0 with E = 1e^-4^) were detected in *Cyanidioschyzon merolae *or *Chlamydomonas reinhardtii*.

**Figure 6:**
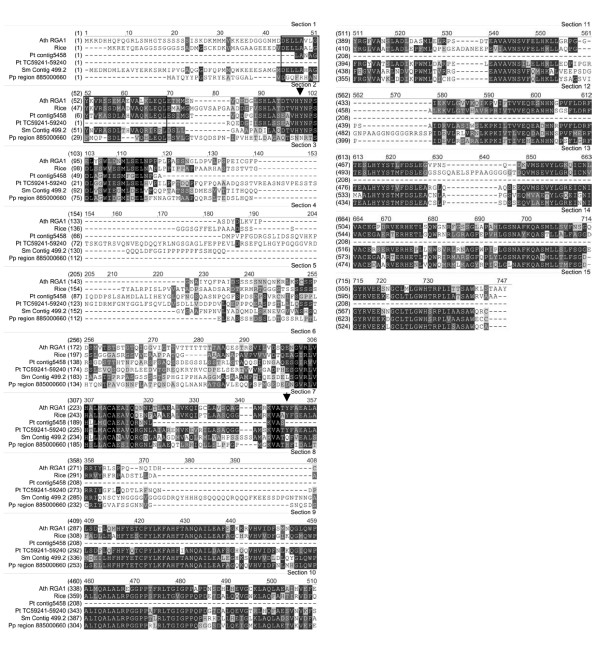
Alignment of the DELLA homologues. The DELLA region appears in the black frame. Ath: *Arabidopsis thaliana*, Sm: *Selaginella moellendorffii*, Pp: *Physcomitrella patens*, Pt: *Pinus taeda*. Arrows indicate structurally important tyrosine residues.

The N-terminal part of *Arabidopsis *RGA1 did not yield a counterpart using tBLASTx against the genome traces nor the ESTs of *Pp*. Reconstruction of the genomic region of the closest RGA1 homologue confirmed the absence of substantial homology in the N-terminal DELLA domain (Figure 6). Instead of the DELLA motif, the *Pp *protein has the amino acids DQGFR. Furthermore, the *Pp *protein lacks two of the four conserved Tyr residues that are structurally important in the *Arabidopsis *DELLA protein RGL2 and that render RGL2 susceptible to GA mediated degradation (Figure 6; [44]).

In contrast, *Selaginella *contains a clear RGA1 orthologue. The protein (Figure 6) was reconstructed from the genomic sequence, since the matching ESTs do not cover the N-terminal part, possibly due to absence of that part in the EST library. Parts of the sequence from the genomic reconstruction, may represent introns, although the EST that covers the C-terminal part (from position 340 onward in Figure 6) has the same sequence as the genomic.

From a limited EST set (~6000) of the fern *Ceratopteris richardii*, we only found a scarecrow-like gene (AY974159) as closest homologue to RGA1. However, as for SLY1, considering the small size of the EST collection we cannot exclude the existence of DELLA orthologues in ferns.

**Figure 7:**
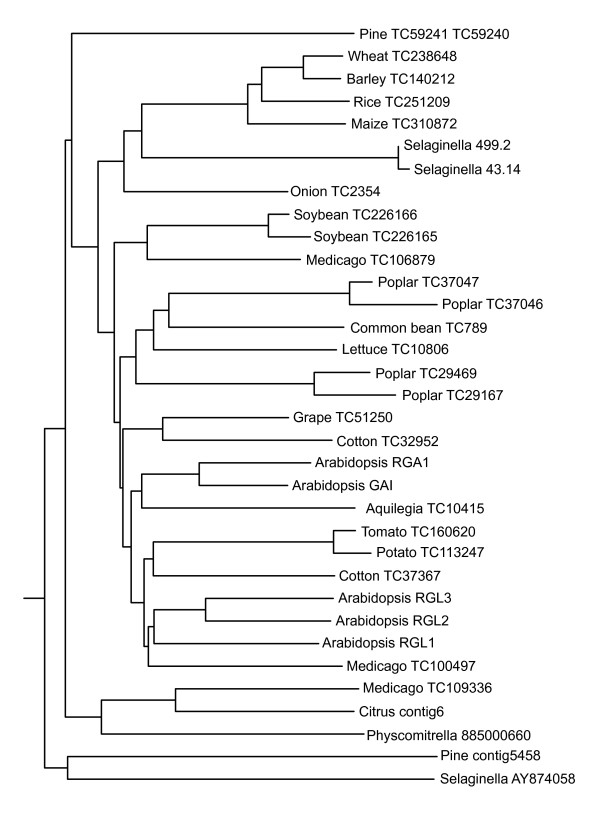
Neighbour-Joining tree of DNA sequences homologous to that encoding the Arabidopsis RGA1 DELLA protein.

Although no complete DELLA protein sequence could be retrieved from the available contigs and ESTs of pine, overlapping homologous fragments from two different proteins suggest the existence of genuine orthologues (e.g. similarity of *Pt *TC59241-59240 with *Ath *RGA1 is 46%). In gymnosperms as well as angiosperms, DELLA orthologues are present in various species (Figure 7). Similarly to the situation of the GA receptors and the F-box proteins, the monocots are more closely related with the gymnosperms and/or *Selaginella*, than the eudicots.

It is noteworthy that there are more ESTs with homology to DELLA coding sequences; however, these lack the N-terminal part spanning the DELLA domain (see Additional file 1). This may be either due to a lack of the 5'end of the coding sequence within the available EST collections or simply because some proteins contain only homology to the C-terminal part [45].

### Effect of exogenous GA treatment on Physcomitrella growth

**Figure 8:**
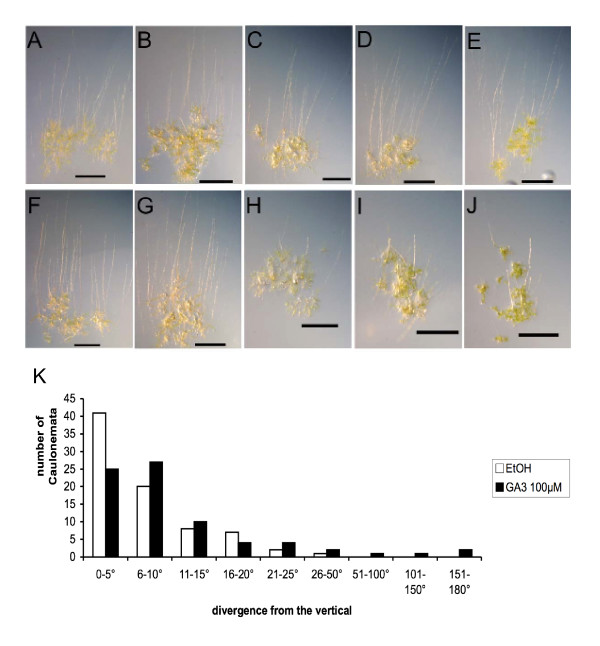
Effect of various concentrations of GA_3 _on caulonema formation and growth direction of *Physcomitrella *protonemata after transfer to darkness and reorientation of Petri dishes into an upright position. A-E) Photographs of mock treated plants as controls for panels F-J respectively. F-J) Photographs of plants treated with 0 μM (F), 10 μM (G), 100 μM (H), 500 μM (I), 1000 μM (J) of GA_3 _(K) Quantification of direction of growth at a treatment of 100 μM GA_3_.

Since GAs are potent growth stimulating hormones in vascular plants, and *Physcomitrella *has at least a protein that is closely related to gibberellin receptors, the question is prompted whether or not *Physcomitrella *can respond to gibberellins. Treatment with 10, 100, 500 and 1000 μM of GA_3 _did not influence general growth of protonemata (comprised of chloronemata and caulonemata; [46]) under normal light conditions. However, when grown in the dark in upright position, the controls as well as plants treated with 10 μM GA developed elongated caulonemata growing positively gravitropic. At relatively high GA_3 _concentrations of 100 μM and more caulonema formation was strongly retarded and growth orientation was changed (Figure 8). While untreated protonemata grow vertically, GA_3 _treated ones grew in various direction showing angles divergent from the vertical, suggesting a defect in gravitropism (Figure 8K). This response is reminiscent of the effect of high concentrations of gibberellin (500 μM) that affect gravitropism in protonemata of another moss, *Ceratodon purpureus *[16].

## Discussion

### Where on an evolutionary scale did the GA signalling pathway arise?

A central node in evolutionary plant research may be the investigation of mosses for which *Pp *stands model [47,48]. GAs are present in various moss species [8]. At present no data on GA content of *Physcomitrella *are available, but homologues to key enzymes (genes GA1-GA5 of *Arabidopsis*) of the GA biosynthetic pathway were found (data not shown). Although *Physcomitrella *has homologues to all three GA signalling components, it is questionable whether they are true orthologues. It is therefore likely that gibberellin signalling does not function in mosses as in vascular plants. Divergence from SLY1 of the F-box protein sequence outside of the F-box, and absence of homology to the "DELLA" region in the closest relative of RGA1, may indicate the existence of other recognition motives between the DELLA-like transcription factor and the SLY1/GID2-like F-box protein. In addition, the DELLA-like protein of *Physcomitrella *may not be functioning as a GA-regulated repressor of GA response as it is stable upon GA treatment when expressed in *Arabidopsis *[13]. It could rather play a regulatory role in plant development, being stable upon GA treatment, similarly to the SLR-like GRAS proteins of rice [45]. Furthermore, since "genuine" GA responses in *Physcomitrella*, e.g. stimulation of growth, are missing, this class of hormones might be less effective in mosses. However, exogenous gibberellins are effective in stimulating growth of seta from the liverwort *Pellia epiphylla*, suggesting the existence of gibberellin signalling earlier in evolution [49].

In conclusion, mosses may have lost the capacity of effective GA signalling or use an alternative system to that of vascular plants to pass on the signal. Whether this involves the homologues to the vascular plant pathway is not known. In the future, it may be interesting to study *Chara*, an even more primitive embryophyte than *Physcomitrella*, which was reported to be GA-responsive [22].

The lycophyte *Selaginella moellendorffii *has clear orthologues for all components of the GA pathway and is to our present knowledge the most primitive species to possess a full-potentially functional – GA signalling pathway; however, phenotypic responses still need to be discovered [13]. Hence, as suggested in earlier studies by its apical growth that is restricted to the sporophyte, the presence of vascular tissue, the formation of roots and leaves, and even by its genome sequences [50,51], *Selaginella *is closely related to seed plants. This is confirmed at the level of the molecular components of GA signalling.

### GA signalling in non vascular plants?

**Figure 9:**
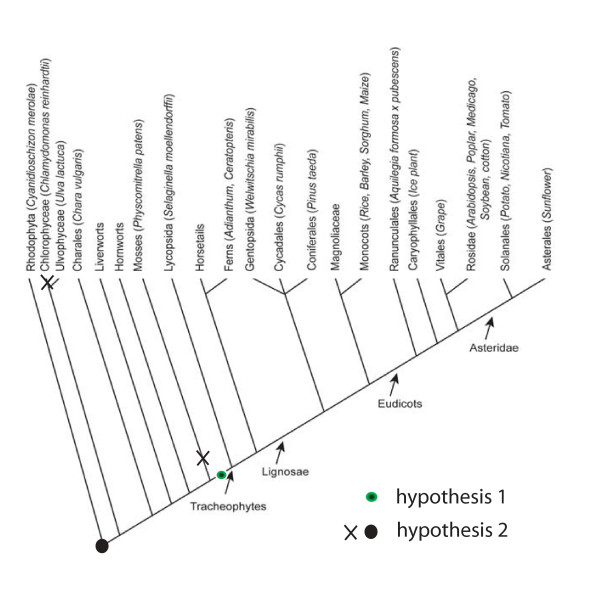
Occurrence of the GA signalling pathway in the plant kingdom. Hypothesis 1: The GA signalling pathway of vascular plants arose after the separation of the Bryophytes. Hypothesis 2: The GA signalling pathway originated earlier than the branching of mosses from the ancestral line, but was lost in unicellular algae and evolved differently in mosses. Dots indicate possible point of appearance of GA signalling. X-marks indicate points of loss of GA signalling.

In recent years, no convincing effect of GA on elongation growth in non vascular plants has been reported. Early publications on GA effects on plants should be read with care, as purity of the compounds used in the treatments was often not that obvious [52], due to lack of modern analytical techniques. Moreover, some of the analyses were done in the presence of high concentrations of other hormones such as auxins or cytokinins [11]. Since in the last two decades, no true hormonal (at concentrations lower than 10 μM GA) effect in other organisms than vascular plants has been reported, it is tempting to speculate that GA signalling has arisen with the occurrence of vascular plants (hypothesis 1 in Figure 9).

If the effects shown in those early studies are indeed true GA effects, then it may be that mosses and other clades lost their capacity to respond to GAs (hypothesis 2 in Figure 9). It is however also possible that non-vascular plants have adopted other mechanisms than those operating in vascular plants to pass on the GA signal, eventually serving for other, yet to be discovered, responses. Assuming that GA signalling as we know it is typical for vascular plants, and that non-vascular species have other compounds and an other mechanism to trigger GA-like responses, a parallel with the evolution of GA biosynthesis may exist. The fungus *Gibberella *has a totally different enzyme set to synthesize GAs than plants [3]. It has therefore been proposed that the synthesis of GAs is a case of convergent evolution in fungi and plants, involving paralogues rather than orthologues.

In ferns, we found homologues to members of the carboxylesterase and scarecrow-like gene families, to which respectively the GA receptors and the DELLA transcription factors belong. Considering the presence of GA responses in ferns [4] and the limited number of EST sequences available, it can be reasonably assumed that in the future, orthologues to GA signalling components will be found.

### Gibberellin as sex determinant

Apart from a possible role in elongation, it is interesting that in non-seed plants as ferns and even Chara, and in some eudicotyledonous plants, male organs are promoted by GAs [23,25]. At this moment it is not known whether the same receptor, F-box, and DELLA components are involved both in elongation growth and in sex determination. The first function of GAs may have been the stimulation of male gametophyte function. It is therefore possible that GA signalling arose as a sex determinant in the first place. The GA receptors were first implemented as part of the carboxylesterase family, showing homology to PrMC3. Intriguingly, PrMC3 was originally isolated from and shown to be expressed specifically in male cones of *Pinus radiata *[53]. Future molecular physiological research is necessary to unravel the importance of this family of carboxylesterases in plants and the reason why some of their members are present especially in (male) reproductive organs.

## Conclusion

The gibberellin signalling pathway as it is known for *Arabidopsis *and rice is well conserved in lycophytes, gymnosperms and angiosperms, which reflects a wide spreading among land plants known to date. However, except perhaps for the receptor, the pathway components seem to be missing in the moss *Physcomitrella*, which indicates that bryophytes may have the evolutionary onset to respond efficiently to gibberellins, which yields other responses (e.g. gravitropism) than in vascular plants. No nuclear EST or genome sequences are available from other non vascular plants and protists such as Charales, nor from multicellular algae, and only limited in ferns. But, a large amount of EST collections and BAC clones of a variety of organisms is awaiting sequencing (CUGI, Clemson, SC). As more genomes will have been sequenced, a more complete picture of the phylogeny of GA signalling will be drawn.

## Methods

### Sequence retrieval

Arabidopsis cDNA sequences from the GID1b, SLY1, and RGA genes were taken from the TAIR website. These sequences were used to search for homologues by tBlastx with default parameters against the unigene collection of Tigr, the *citrus *HarvEST database (Wanamaker and Close, University of California), the Cosmoss transcriptome and genome (with an eight times sequencing coverage) databases (Stefan Rensing, University Freiburg, Germany), the PHYSCOBASE database [54], the *Selaginella *databases at Purdue University [55], the *Cyanidioschyzon merolae *database of Tokyo University [56], the loblolly pine ESTs from the CCGB of the University of Minnesota, the *Cycas *EST collection from the New York Botanical Garden and the sequenced EST collection of *Adiantum capillis veneris*, *Marchantia polymorpha*, *Welwitschia mirabilis*, *Ceratopteris richardii*, *Mesostigma viridae *gathered at plantGDB. Tables with reference numbers can be found in supplemental data (see Additional files 1, 2, 3).

### Reconstruction of protein sequences

Retrieved ESTs from *Selaginella moellendorffii *were used to scan the Sellaginella genome for homologous sequences. 5' and 3' ends were taken to virtually walk over the chromosomes and isolate clones covering about 10 kbp around the original EST (which is homologous to the *Arabidopsis *sequence). For the *Physcomitrella patens *F-box protein, the same approach was used. For the the DELLA homologue, the *Arabidopsis *protein sequences were directly used to scan the genome traces of the Cosmoss dbase (Stefan Rensing, University Freiburg). The retrieved clones (see Additional files 4, 5, 6) were submitted to a PHRAP process to yield contigs. From these contigs, proteins were derived by doing an *in silico *translation [57]. The *Pp *F-box protein homologue and DELLA homologue translated as a single exon from the Cosmoss database. A *Pp *receptor homologue had a predicted intron between R157 and R158 (Figure 2). The predicted intron was left out in the reconstructed protein and was 2 bp out of frame at the 3'end. For the aligment in Figure 2, the sequence of Physcobase contig 12981 was used. *In silico *translation was also applied to the ESTs from pine. Open reading frames were detected and checked for homology with the original *Arabidopsis *proteins in a BlastP.

### Alignments

Protein alignments were performed using ClustalW algorithms included in the VectorNTI package (Invitrogen). Multiple alignments of coding sequences and ESTs were done on line, using ClustalW at [58] (Kyoto University Bioinformatics Center) using default parameters and choosing the PHYLIP output. Neigbour-Joining trees were generated at [58]. Various control runs were done using IUB (at [58]), Dialign in the Panta rhei package [59]; University of Bielefeld, Germany) and MAVID [60] alignment protocols. All controls yielded similar results. Similarity values were calculated with VectorNTI software (Invitrogen).

### Gibberellin treatments of Physcomitrella

*Physcomitrella patens *(Hedw.) B.S.G. was cultured in liquid and solid Knop medium as described earlier [61]. GA_3 _was purchased by Duchefa (Haarlem, The Netherlands) and a 0.25 M stock solution was prepared in EtOH. For the treatments 5 μl of freshly subcultured moss protonema in liquid culture with a dry weight of 100 mg/l were placed on Knop plates containing 0, 10, 100, 500 and 1000 μM GA3 or control plates containing the corresponding amount of EtOH. After one week of growth at normal conditions (25°C, 16 h light with 55 μmol/sm^2^, 8 h darkness) the plates were covered with aluminium foil and put upright for 7 days. For documentation the plants were photographed.

## Abbreviations

GA: gibberellin

Pp: Physcomitrella patens

Sm: Selaginella moellendorffii

GID1: gibberellin insensitive dwarf, a GA receptor

SLY: SLEEPY, an F-box protein involved in the GA signalling pathway

RGA: Repressor of *ga1*; one of the DELLA proteins, acting as a repressor of GA response

## Authors' contributions

FV and DVDS conceived the study. FV retrieved the sequences and performed the alignment assays. ACF helped in the assembly of sequences and reconstruction of contigs. GW performed the physiological tests on *Physcomitrella patens*. FV wrote the manuscript together with RR and DVDS. All authors read and approved the final manuscript.

## Supplementary Material

Additional file 1 This file contains a list of the TIGR contigs (gene indices), homologous to *Arabidopsis thaliana RGA*, used in the evolutionary trees with indication of the organism. Click here for file

Additional file 2 This file contains a list of the TIGR contigs (gene indices), homologous to the *Arabidopsis thaliana *GID1 GA receptor, used in the evolutionary trees with indication of the organism. Click here for file

Additional file 3 This file contains a list of the TIGR contigs (gene indices), homologous to *Arabidopsis thaliana SLEEPY1*, used in the evolutionary trees with indication of the organism. Click here for file

Additional file 4 This file contains the numbers of the ESTs and contigs used for reconstructing the Selaginella and Physcomitrella homologues of the *Arabidopsis thaliana *GA receptor GID1. Click here for file

Additional file 5 This file contains the numbers of the ESTs and contigs used for reconstructing the Selaginella and Physcomitrella homologues of the *Arabidopsis thaliana SLEEPY1 *gene. Click here for file

Additional file 6 This file contains the numbers of the ESTs and contigs used for reconstructing the Selaginella and Physcomitrella homologues of the *Arabidopsis thaliana RGA *gene. Click here for file
